# The Examination of Proposers for Life Insurance

**Published:** 1914-07-25

**Authors:** 


### THE PROFESSION OF MEDICINE.

### The Examination of Proposers for Life Insurance.

JUST lately the efficacy of the preliminary medical examination of those who propose to insure their lives has been called in question in the lay press. It has been alleged that this routine examination, on which all but one or two companies still insist, is unnecessary and useless; that it results in the refusal of good lives and the acceptance of bad ones; and, in fact, that no harm would come of abandoning it altogether because it is inefficient and costs money. Now these allegations, unsound and refutable as we believe them to be, affect the medical profession, or no small portion of it. For very large numbers of practitioners undertake medical examinations for insurance companies, and derive a part of their incomes from this source; and the agitation in question not only threatens them in pocket, but is also an imputation on their professional ability and acumen.

Let it be admitted that cases have occurred of proposers being refused, or at least rated otherwise than as first-class lives and "loaded" accordingly, who have subsequently falsified the implied prognosis by living to a green old age. Admittedly also proposers have been accepted as first-class lives who have shortly afterwards developed serious constitutional diseases and died of them. Such cases can never be eliminated entirely until medicine becomes as exact a science as mathematics. The real point at issue is not whether such errors are or are not made, but whether they bear any considerable proportion to the cases in which no error is made. We make bold to say, both from statistical and from personal experience, that they do not; that of those refused as bad lives only a small fraction live long to refute the judgment of the medical examiner, and that of those accepted only a very minute proportion indeed are actually suffering at the time from any disorder which could be or ought to be detected by medical examination. We hold that the insurance companies derive a very definite and very appreciable protection through the medical examination on which they insist, and that the extremely able and astute managers of those companies would long ago have abandoned this precaution were they not convinced of its value.

### THE ROMANCE OF INSURANCE.

There is a species of romance about some of the numerous strange incidents of the insurance medical officer's experience. Few men who have held such a post for years could not tell strange stories of their interesting cases. Sir John Collie, whose experience of all kinds of medical examination work is enormous, told a good story some time ago to illustrate the need of perpetual vigilance. A certain man was insured in a life office as a first-class life, and after paying his premiums regularly for some time, he asked to have his insurance very greatly increased. In such a case a re-examination is always necessary, for otherwise a man who happened to develop any serious disease—say, diabetes, Bright's disease, or cancer, for example—might thus defraud the insurance company. The man was accordingly instructed to attend before the insurance company's medical officer, which he did. In the usual way he was requested to strip to the waist, and the ordinary examination of his heart, lungs, and urine was conducted. The man was a well-developed specimen physically, and striking an attitude before the doctor exclaimed somewhat dramatically, "Am I not a fine fellow?" or words to that effect. The doctor was a little amused at this conceited speech, but finding nothing abnormal in his investigations, passed the man as a good life. Three or four months later the man died of cancer of the rectum, and his representatives claimed the full amount of the increased assurance. The insurance company was naturally somewhat startled by a heavy claim in respect of a man passed by the doctor as sound a short time before, and inquiries were set on foot. It then turned out that at the very time of the examination the man had a colostomy opening, performed not long before for intestinal obstruction from the growth, and his theatrical outburst before the doctor was a device to distract attention from the lower part of his abdomen, which he had of course kept carefully concealed.

### THE SEEDS OF DISEASE.

Such cases, however, are rare, and it is very seldom that imposture of this sort is successful. On the other hand, it is not at all uncommon for the medical examiner to detect signs of incipient serious disease for which the proposer may not have yet bothered himself to procure treatment, or of which he may even be quite unaware. The present writer can quote two such cases from his own experience, and probably everyone who does much insurance work could duplicate them. One was the case of a young man who proposed himself for life insurance, believing himself to be in perfect health. His urine was found to contain albumin, which was persistent and could not be said to conform to the comparatively harmless type of functional albuminuria. To cut a long story short, he soon afterwards developed frequency of micturition, and expert investigation revealed the fact that he had tuberculosis of the kidney. The other was a man of about forty, who also believed himself in perfect health, and had not consulted a doctor since he had been grown up. He had a trace of albumin in a pale urine, together with a considerable enlargement of the heart. He was refused on the score of incipient chronic Bright's disease, and advised to see his own doctor about it. He accordingly went to his doctor, who tested a sample of urine and found no albumin. The doctor then wrote to know on what grounds the diagnosis had been made, giving his reason for disagreeing with it. He had not, it turned out, noticed the cardiac enlargement and hypertrophy, and he was also advised to test a twenty-four hours' sample. He did this, found albumin, concurred in the diagnosis, and put the patient on the diet and regimen necessary to put off for as long as possible the inevitable breakdown of the patient's health.

### Assurance Without Medical Examination.

These two cases supply the answer to another point which those who decry medical examination frequently raise. They say that there are life offices—as indeed there are one or two—which dispense with the medical examination and yet do not go bankrupt from accepting injured lives. It is true that these offices get along fairly well, although it is certain that better selection of lives by proper medical examination would result in the exclusion of some damaged lives which they at present accept. There are two reasons why they get along fairly well. In the first place they secure a large number of proposals from people who, though perfectly sound lives, shrink from what they regard as the ordeal of a medical examination. That is to say, the omission of medical examination brings them a lot of business which they would otherwise not get. And in the second place they put very searching questions to all proposers regarding past illnesses and refusals by other offices, thereby protecting themselves against a good many "crocks" and against those whom the medical officers of other companies have already rejected. Prevarication over the answers to such questions invalidates the contract of insurance, thus preventing the mere liar from imposing on the company. It will be obvious, however, that both the proposers whose cases have been described above would have been accepted, for neither of them had the slightest idea that he was in impaired health, though each was in the very early stage of an insidious malady which had not yet attracted his attention by causing noticeable symptoms. And it follows that companies which dispense with medical examination must insure more bad lives unwittingly than those which do not dispense with it, even though the proportion may be small. Add to this, that their officers must be perpetually on the watch for fraud, never easy to prove when the suspected person is already defunct, and it is easy to understand why the great majority of the offices retain their faith in medical examination.

### Effect of Medical Progress on Premiums.

As a matter of fact, the competition for business among the offices is so strong nowadays that slight deteriorations of health are often passed without penalising the proposer, such as would a generation ago have involved heavy "loading" of the premiums; and quite fairly formidable degrees of impairment are accepted at "loaded" rates, which would formerly have entailed complete rejection. Part of the reason for this is, no doubt, the progress of medical science, which has discovered so many means for curing disease and prolonging life unknown a generation ago. Thus the prognosis of many chronic diseases is much better, and can be much more confidently made now than it was a few years ago, and hence the offices are able to appreciate and to assess much more exactly the risk which they run in accepting proposers exhibiting them. But this, it need hardly be said, is no argument against medical examination: rather the reverse.

